# Risk of Endophthalmitis in Boston Type 1 Keratoprosthesis Combined with Vitrectomy and Silicone Oil Insertion

**DOI:** 10.1155/2019/9648614

**Published:** 2019-07-25

**Authors:** Mohamed Abou Shousha, Taher Eleiwa, Allister Gibbons, Christopher Smith, Sean Edelstein, George Kontadakis, Zachary Schmitz, Joshua Abernathy, Ross Chod, Zachary Bodnar, Kelvin McDaniel, Rocio Bentivegna, Levent Akduman

**Affiliations:** ^1^Saint Louis University Eye Institute, St Louis, MO, USA; ^2^Bascom Palmer Eye Institute, Miami, FL, USA; ^3^Department of Ophthalmology, Faculty of Medicine, Benha University, Benha, Egypt

## Abstract

**Purpose:**

To identify the incidence of endophthalmitis and visual outcomes in eyes with Boston type 1 keratoprosthesis combined with pars plana vitrectomy and silicone oil insertion (KPro + PPV + SOI) as compared to eyes receiving Boston type 1 keratoprosthesis (KPro) alone.

**Patients and Methods:**

Retrospective chart review of 29 eyes of 27 patients with KPro having at least 12-month follow-up. Thirteen of these eyes had hypotony and/or retinal detachment in addition to corneal pathology and thus received KPro + PPV + SOI. Polymyxin-trimethoprim with a quinolone was used as chronic topical antibiotic prophylaxis in both groups after the first postoperative month. Outcome measures recorded at the 1-, 3-, 6-, 12-, and 24-month follow-up visits included best-corrected visual acuity (BCVA) and rates of postoperative complications.

**Results:**

All the patients had completed 24-month follow-up except one case in the KPro group who lost to follow-up after 12-month visit. In the KPro + PPV + SOI group, no eyes had developed endophthalmitis by the 24-month follow-up visit versus 5 eyes of 5 patients in the uncombined KPro group (*P*=0.048). The 2-year cumulative endophthalmitis incidence was 31.2% in the KPro group versus zero in the KPro + PPV + SOI group (*P*=0.030). Four of these 5 eyes had vitreous taps with positive cultures; 2 were positive with *Staphylococcus aureus*, 1 with coagulase-negative staphylococci, and 1 with *Streptococcus pneumoniae*. Other complications included KPro extrusion (1 in each group), retinal detachment (2 in the KPro and 1 in the KPro + PPV + SOI group), newly developed glaucoma (2 in each group), and retroprosthetic membrane (9 in the KPro and 5 in the KPro + PPV + SOI group). The KPro group had better average preoperative BCVA compared to those of the KPro + PPV + SOI group (−2.29 ± 0.72 LogMAR, versus −2.95 ± 0.30 LogMAR; *P*=0.004). No statistically significant difference in BCVA was noted in subsequent follow-up visits.

**Conclusion:**

The addition of PPV and SOI to the KPro implantation in the eyes with corneal pathology, as well as hypotony and/or retinal detachment, is a safe and effective procedure for visual rehabilitation. Pars plana vitrectomy and silicone oil insertion may have a protective effect against the development of postoperative endophthalmitis in eyes receiving KPro.

## 1. Introduction

The Boston type I keratoprosthesis (Massachusetts Eye and Ear Infirmary, Boston, MA; KPro) is the most widely used prosthetic corneal transplant in the United States and the world [1]. KPro has gained popularity over the last decade. The number of KPro procedures has increased from fewer than 50 in 2002 to more than 1150 in 2009 [2]. The goal of using a KPro is to attempt to restore vision in patients who would otherwise have a very poor prognosis with penetrating keratoplasty. This subset of patients includes those with previous graft failures, limbal stem cell deficiency, cicatrizing diseases, and chemical injuries [2, 3].

Outcomes of KPro implantation have been encouraging. Nevertheless, long-term studies have shown a high rate of sight-threatening complications with a device retention rate of only 67% at 7 years, highly dependent on the KPro indication [4]. One catastrophic complication of KPro is infectious endophthalmitis. Long-term studies have shown a 7-year cumulative endophthalmitis incidence up to 15.5% after Boston type 1 KPro [4, 5] versus a 5-year cumulative incidence of 1.3% for bleb-related endophthalmitis [6] and 6.3% of endophthalmitis after glaucoma drainage device insertion [7], with both sharing the ongoing risk of infection and worse visual outcomes compared to infectious endophthalmitis after penetrating keratoplasty and cataract surgery. The KPro is a device, with limited biointegration, that bridges a nonsterile ocular surface with a sterile anterior chamber and can lead to rapid invasion of pathogenic organisms through the space between the tissue and the prosthesis [8].

In this study, we report our observation that patients with KPro combined with pars plana vitrectomy and silicone oil insertion have a lower incidence of infectious endophthalmitis than those with KPro alone.

## 2. Materials and Methods

This was a retrospective chart review of patients who underwent KPro implantation and patients who underwent KPro implantation in combination with pars plana vitrectomy (PPV) and silicone oil insertion (SOI) in Saint Louis University Eye Institute (SLUEI) and Bascom Palmer Eye Institute from January 2011 until January 2018. This study was approved by Saint Louis University and the University of Miami Institutional Review Board. Indications for KPro transplantation are listed in Table 1. Eyes that had hypotony and/or retinal detachment in addition to corneal pathology received KPro implantation combined with PPV and SOI. Implantation of KPro in all cases was performed by previously published techniques [9]. Patients, who planned to undergo PPV and SOI, initially underwent an Eckhardt temporary keratoprosthesis implantation followed by PPV and SOI by a retina specialist and then replacement of Eckhardt keratoprosthesis with KPro; all procedures were performed in the same session.

Best-corrected distance visual acuity (BCVA), intraocular pressure, slit lamp examination, and complications were reviewed at the 1-, 3-, 6-, 12-, and 24-months postoperative follow-up visits. The start date of the follow-up was the date of KPro insertion, and the end date was the last date the patient was seen by the ophthalmologist, or KPro removed, or the patient lost all vision in the KPro eye. The patient should have at least 12-month follow-up to be included in the study. Endophthalmitis, visual outcomes, and other complications were compared between groups.

Statistical analysis with SPSS software version 20.0 (SPSS, Chicago, IL, USA) was performed to calculate descriptive statistics for all eyes. Rates of complications were compared between groups by means of chi-squared test. Average visual acuity and duration of follow-up were compared between both groups with two sample *t*-tests. Average visual acuity was compared at different follow-up visits for the endophthalmitis cases using repeated measures analysis of variance (ANOVA). The time-related cumulative incidence of endophthalmitis in each category was evaluated by means of Kaplan–Meier survival curves. Two-sided *p* values less than 0.05 were considered statistically significant. Values are presented as means ± standard deviation.

## 3. Results

The study included 29 eyes of 27 patients. Sixteen eyes underwent only KPro implantation (KPro group), and 13 eyes underwent KPro implantation combined with PPV and SOI (KPro + PPV + SOI group). The mean follow-up was 23 months (range 12–24 months) in the KPro group, versus 24 months in the KPro + PPV + SOI group (*P*=0.377), with a 93.75% of KPro group and 100% of KPro + PPV + SOI group completing 24-month follow-up.  Table 2 summarizes the different features of both groups. The early postoperative management (1 month) included the use of polymyxin B sulfate and trimethoprim (Polytrim, Allergan Inc., Irvine, CA) and ofloxacin 0.3% (Ocuflox, Allergan, Irvine, CA) or moxifloxacin 0.5% (Vigamox, Alcon Inc., Fort Worth, TX) in both groups. This was in addition to topical steroids in both groups; the KPro + PPV + SOI group received dexamethasone and tobramycin (Tobradex; Alcon Labs, Fort Worth, TX), while the KPro group received prednisolone acetate 1% (Pred Forte; Allergan, Irvine, CA). All drops were administered four times daily. Past the 1-month follow-up visit, all patients were kept chronically on one regimen, polymyxin B sulfate and trimethoprim (Polytrim, Allergan Inc., Irvine, CA) and ofloxacin 0.3% (Ocuflox, Allergan, Irvine, CA) or moxifloxacin 0.5% (Vigamox, Alcon Inc., Fort Worth, TX) administered twice daily. Vancomycin-fortified eye drops were not prescribed for prophylaxis due to availability and cost issues. Four patients in the KPro group and 6 patients in the KPro + PPV + SOI group did not use contact lenses chronically secondary to intolerance. There were no persistent epithelial defects or infectious keratitis of the corneal carrier tissue noted in any of the included cases.

**Figure 1:**
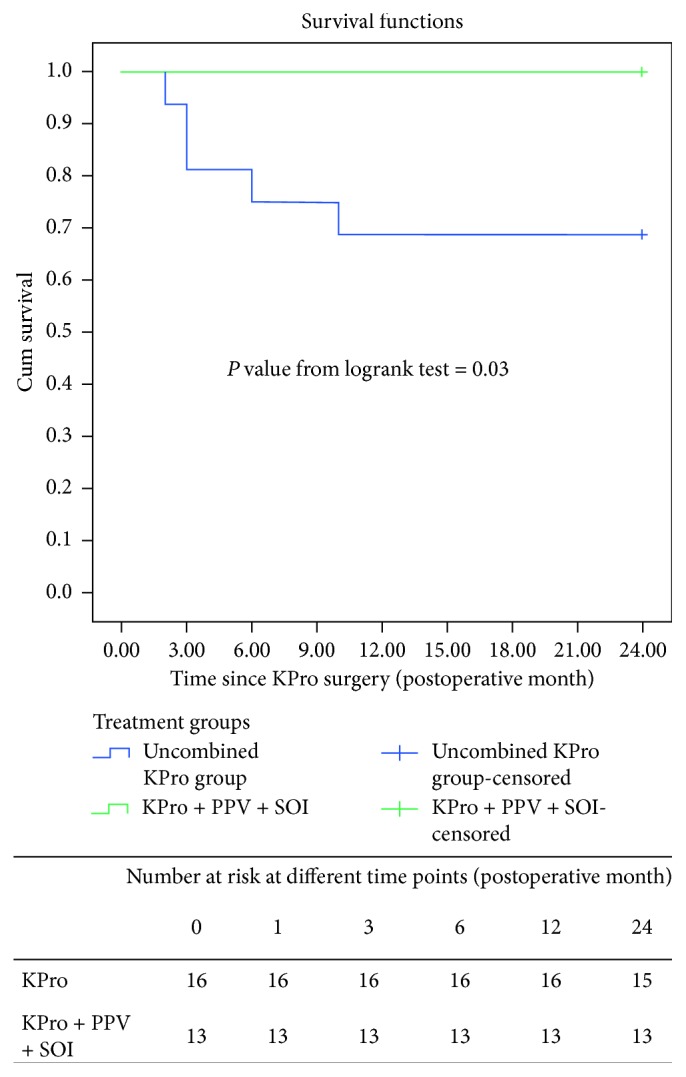
Kaplan–Meier graph of the incidence of endophthalmitis in the Boston type 1 keratoprosthesis (uncombined KPro) group versus combined KPro, pars plana vitrectomy, and silicone oil insertion group (KPro + PPV + SOI), with the number of subjects at risk at different time points listed underneath the figure. The 2-year cumulative (Cum) survival ratio was 68.8% in the KPro group, versus 100% in the KPro + PPV + SOI group (*P*=0.03).

**Figure 2:**
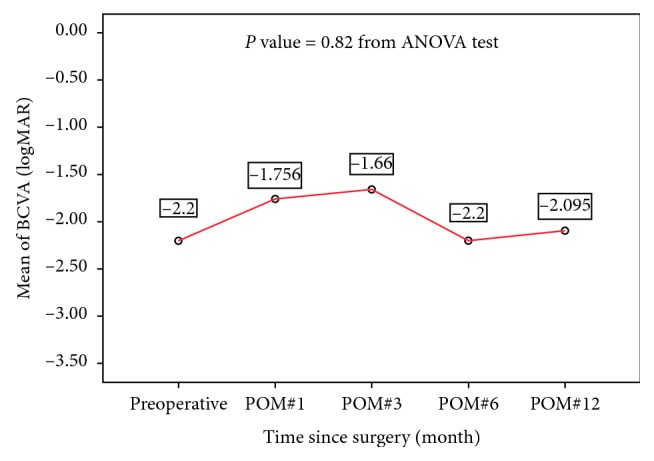
Graph illustrating visual performance in endophthalmitis cases at different follow-up visits. POM: postoperative month; ANOVA: analysis of variance.

During the 24-month follow-up period, no eyes in the KPro + PPV + SOI group developed endophthalmitis versus 5 eyes of 5 patients in the KPro group (*P*=0.048, Fisher's exact test).  Table 2 summarizes the characteristics of the 5 cases. Endophthalmitis occurred within a range of 2–10 months. All the cases were compliant with their topical antibiotic drops at the time of onset of endophthalmitis. The Kaplan–Meier analysis for the incidence of endophthalmitis in the KPro versus KPro + PPV + SOI groups is given in Figure 1. Among those managed with the KPro + PPV + SOI procedure, the incidence of endophthalmitis was zero at two-year follow-up, while the uncombined KPro group had a 2-year cumulative incidence ±SE of endophthalmitis of 31.2% ± 11.6% (*P*=0.030). Patients who developed endophthalmitis received an intravitreal tap and injection of antibiotics. Vitreous aspirate cultures were positive in 4 cases: *Staphylococcus aureus* (2 cases), coagulase-negative staphylococci (1), and *Streptococcus pneumoniae* (1). There was no significant difference in the rate of endophthalmitis between patients who used contact lenses chronically versus those who did not. The KPro was not removed in the 5 cases, and they maintain potential vision till the last date of follow-up (Table 3). Only one case was lost to follow-up after the 12-month visit.  Figure 2 demonstrates the visual performance in endophthalmitis cases.

**Figure 3:**
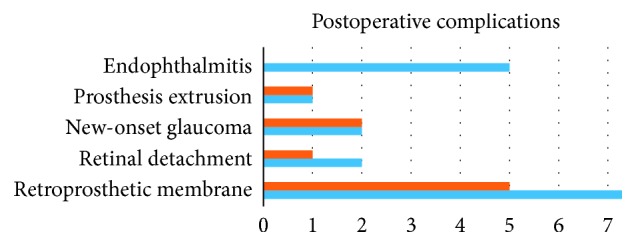
Graph illustrating the postoperative complications in the Boston type 1 keratoprosthesis (KPro) group versus the combined KPro, pars plana vitrectomy, and silicone oil insertion group.

**Figure 4:**
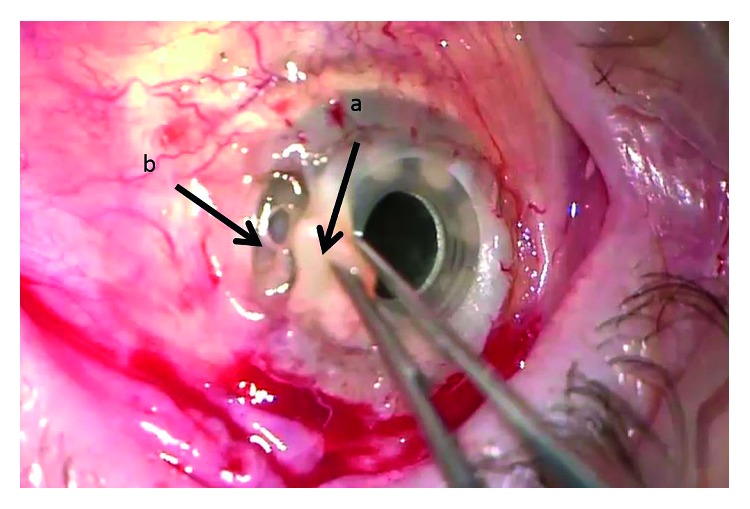
Figure depicting the melting corneal graft (a) around the Boston type 1 keratoprosthesis (KPro) of patient number #1 in the KPro combined with the pars plana vitrectomy and silicone oil insertion group. Despite that the posterior plate of the KPro (b) is fully exposed, there is no leakage of aqueous humor or the intraocular silicone oil, secondary to the presence of a strong retroprosthetic membrane.

Other complications that occurred among both groups are seen in Figure 3. Each group had one KPro extrusion, as well as 2 newly developed glaucoma (*P*=1.0). Retroprosthetic membranes were common among each group, 9 occurring in the KPro and 5 in the KPro + PPV + SOI group (*P*=0.340). Two of the patients in the KPro versus 1 in the KPro + PPV + SOI group had retinal detachment (*P*=1.0). Nine of the patients in the KPro-only group had a glaucoma drainage implant (GDI) versus only one in the combined group (*P*=0.008). Two of those patients with the GDI developed endophthalmitis without evidence of GDI infection. There was no statistically significant difference between incidences of endophthalmitis in patients with or without GDI in our case series (*P*=0.549). One of the patients in each group had a corneal melt that led to extrusion, yet it was noted that the retroprosthetic membrane was very dense and there was no leakage of the intraocular contents (Figure 4).

**Figure 5:**
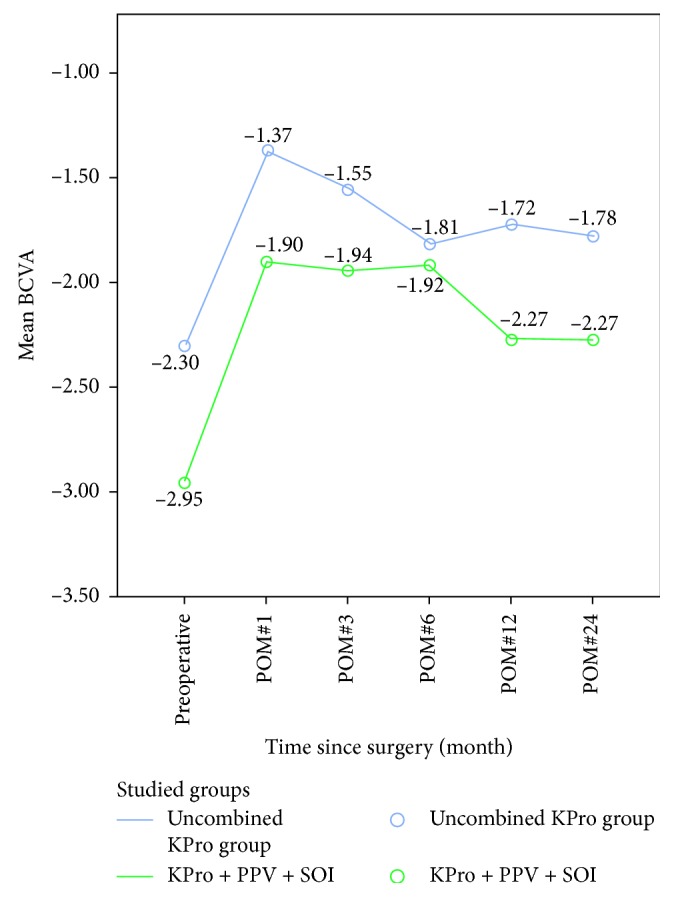
Graph illustrating the difference in the BCVA between the KPro group and the KPro + PPV + SOI group; BCVA preoperatively in the KPro group was −2.29 ± 0.72 LogMAR and −2.95 ± 0.30 LogMAR in the combined group (*P*=0.004, *t*-test). After the surgery, BCVA was not statistically significant between each group.

The KPro-only group had better average preoperative BCVA as compared to those of the KPro + PPV + SOI group (Figure 5). BCVA preoperatively in the KPro group was −2.29 ± 0.72 LogMAR (Snellen equivalent of 1/200) versus −2.95 ± 0.30 LogMAR (Snellen equivalent of HM) in the combined group (*P*=0.004, *t*-test). After the surgery, BCVA was not statistically significant between each group.

## 4. Discussion

The results of this study suggest that pars plana vitrectomy and silicone oil placement could lower the rate of endophthalmitis in eyes with Boston type 1 keratoprosthesis. Boston type 1 keratoprosthesis provide the ability to restore vision in selected patients with corneal blindness when a corneal transplant is estimated to have a worse outcome. As with any keratoplasty, this is associated with an increased risk of endophthalmitis [10]. Between the years of 1999–2009, one large academic institution published a rate of endophthalmitis from all intraocular surgeries to be 0.065% and from cataract surgery to be 0.041% [11]. Bleb-related endophthalmitis is the second most frequent cause of postoperative endophthalmitis after acute and chronic postcataract surgery endophthalmitis [12]. Its incidence is reported to be between 0.2% and 1.3%, [13, 14] and increases with the use of antiproliferative agents (up to 3%) and with inferiorly placed blebs (up to 9.4%) [15–18]. Both bleb and KPro-related endophthalmitis share the same ongoing risk of infection, virulent organisms crossing an altered barrier between the ocular surface and the aqueous, late onset endophthalmitis and poor visual outcome [19]. Taban et al. reported a rate of endophthalmitis of 0.382% for penetrating keratoplasty [20]. Rishi et al. reported an incidence of 9% over a ten-year follow-up period with Boston keratoprosthesis type 1 [21]. As discussed earlier, the rate of endophthalmitis in patients with KPro has been reported to be as high as 15.5% over a 7-year follow-up period [4, 5]. In this study, the overall incidence of endophthalmitis within the follow-up period was 17%; however, it was higher in the uncombined KPro group. Grassi et al. reported that sterile vitritis after KPro can mimic infectious endophthalmitis with pain and external signs of inflammation [22]; however, the absence of overt clinical signs does not rule out infection either [23]. The prognosis also appears to be worse when endophthalmitis occurs after KPro implantation, versus other surgeries [5]. Endophthalmitis isolates in our study were all Gram-positive. Gram-positive bacteria were found predominant in endophthalmitis [10, 19, 24–27]. No susceptibility data were obtained in our study. However, according to the literature, Gentile et al. reported susceptibility to fluoroquinolones for Gram-positive isolates from a low of 65% for levofloxacin to a high of 78% for gatifloxacin, versus 99.7% to vancomycin during the time period from 1987 to 2011 [24]. Durand et al. reported a lower incidence of bacterial endophthalmitis (1%) in KPro eyes with prophylactic topical vancomycin, given its excellent Gram-positive coverage [19]; however, it was not prescribed in this study for prophylaxis due to availability and cost issues.

In our study, the 2-year endophthalmitis rate was lower when KPro was combined with pars plana vitrectomy and intraocular silicone oil (*P*=0.03). One possible mechanism is that silicone oil has antimicrobial properties. Özdamar et al. tested the antimicrobial properties by incubating silicone oil with several known endophthalmitis-associated microorganisms. Results showed a decrease in growth in all of the colonies incubated with silicone oil [28]. Another mechanism may be that silicone oil leads to the formation of a strong retroprosthetic membrane that acts as a mechanical barrier to pathogenic organisms. An image of this membrane can be seen in Figure 4. While, we understand that silicone oil insertion has its own complications and that it is not recommended to place silicone oil for mere prophylaxis, we believe that our observation has the potential to direct future research in the development of new keratoprosthesis to avoid the unacceptable high rate of postoperative complications.

Silicone oil is used in order to facilitate intraocular tamponade. It is thought to prevent uveal edema and hypotony by maintaining long-term pressure in the eye. This avoids retinal and choroidal detachments from occurring [29]. In our cases, silicone oil placement was used to treat hypotony and retinal detachment. Silicone oil placement was combined with KPro due to a history of multiple graft failures, or a very low success rate anticipated with a traditional corneal graft, given severe hypotony and a need for chronic silicone tamponade in prephthisical eyes. Utine et al. and Chan et al. had reported on the use of KPro + PPV + SOI for the visual rehabilitation of chronic hypotony and corneal opacity [30, 31]. Both these case series are in agreement with our study as they did not indicate that any of their patients developed endophthalmitis. Additionally, Iyer et al. reported a zero incidence of endophthalmitis in 40 silicon oil-filled eyes who underwent keratoprosthesis with a mean follow-up of 61.54 months for Boston type 1 KPro [32]. On the other hand, RPM was the most common complication (42.5%) [32]. Our study is the first to specifically investigate the effect of combining KPro with PPV and SOI on the rate of endophthalmitis and showed that cases that underwent KPro + PPV + SOI had a significantly lower rate of endophthalmitis compared to those who underwent KPro implantation alone.

Regarding visual outcomes, there was no statistically significant difference in BCVA between both groups during the postoperative visits. This is likely related to a selection bias as those patients who received the KPro + PPV + SOI had worse visual acuity preoperatively. And as complications occurred, both groups tended to equalize. Another possible explanation for this is that silicone oil/KPro posterior plate interface induces an unanticipated high hyperopic error of refraction [33]. Further studies are needed to better describe the effect of the interface on refractive errors.

The limitations of this study include the retrospective study design, the small sample size, and the short follow-up period. The difference in the postoperative management was due to different preferences of the two involved surgeons. However, it is noteworthy that endophthalmitis occurred with both prophylaxis regimens. Also, we reported higher rates of endophthalmitis in the uncombined KPro group compared to the zero incidence in the combined one that could be attributed to the small sample size and the short follow-up period. A randomized prospective multicenter study with longer follow-up would be useful to further evaluate the risk to benefit ratio of the use of silicone oil in KPro patients and its role in preventing endophthalmitis and retinal complications.

In conclusion, the main outcome of our study is that PPV and SOI may have a protective effect against endophthalmitis, a vision-threatening complication not uncommon with Boston type 1 keratoprosthesis. To our knowledge, this is the first time that such effect has been demonstrated in the literature. Larger studies might further elucidate the clinical significance of this finding.

## Figures and Tables

**Table 1:** Indications for surgery.

Boston type 1 keratoprosthesis group (*N*=16)		Boston type 1 keratoprosthesis combined with vitrectomy and silicone oil insertion group (*N*=13)	
Indication	Total	Indication	Total
Multiple graft failures	10	Multiple graft failures and retinal detachment	4
Aniridia and limbal stem cell deficiency	3	Severe alkaline injury, graft failure, and retinal detachment	3
Scleroderma and multiple graft failure	1	Chronic uveitis with hypotony	2
Herpetic keratitis and neurotrophic ulcer	1	Hypotony and graft failure	2
Stevens–Johnson syndrome	1	Herpetic keratitis, neurotrophic ulcer with retinal detachment	1
		Aniridia, limbal stem cell deficiency, and hypotony	1

**Table 2:** Characteristics of the studied groups.

		Studied groups	
		KPro	KPro + PPV + SOI
Age (mean ± SD)		61 ± 18 years	56 ± 25 years
Follow-up (mean; range)		23 (12–24) months	24 months
Number (%) with follow-up to 6 months		16 (100)	13 (100)
Number (%) with follow-up to 12 months		16 (100)	13 (100)
Number (%) with follow-up to 24 months		15 (93.75)	13 (100)
Number (%) with glaucoma drainage implant		1 (7.69)	9 (56.25)
Number (%) wearing bandage contact lenses		12 (75)	7 (65.5)
Number (%) using prophylactic topical antibiotic regimen	Polytrim + ofloxacin 0.3%	9 (56)	7 (54)
	Polytrim + moxifloxacin 0.05%	7 (44)	6 (46)

**Table 3:** Characteristics of endophthalmitis cases.

	Underlying disease	BCVA before endophthalmitis (Snellen)	Time to endophthalmitis (postoperative month)	BCVA after endophthalmitis (Snellen)	End of follow-up (postoperative month)	Prophylactic topical antibiotic	Compliance at time of onset of endophthalmitis	Presence of BCL	Presence of GDI	Microbiology
Case 1	Multiple graft failures	6/200	6	HM	24	Polytrim + moxifloxacin 0.05%	Compliant	Yes	Yes	Negative
Case 2	Aniridia, limbal stem cell deficiency	20/400	2	20/400	24	Polytrim + ofloxacin 0.3%	Compliant	No	No	*Staphylococcus aureus*
Case 3	Herpetic keratitis, neurotrophic ulcer	20/400	10	20/300	24	Polytrim + moxifloxacin 0.05%	Compliant	No (lost in 6 months)	No	Coagulase-negative staphylococci
Case 4	Multiple graft failures	1/200	3	HM	24	Polytrim + ofloxacin 0.3%	Compliant	No (lost after 1 month)	Yes	*Streptococcus pneumoniae*
Case 5	Multiple graft failures	Counting fingers at 3 feet	3	Light perception	12	Polytrim + ofloxacin 0.3%	Compliant	Yes	No	*Staphylococcus* aureus

BCVA: best-corrected visual acuity; BCL: bandage contact lens; GDI: glaucoma drainage implant; HM: hand movement.

## Data Availability

The datasets used and/or analyzed during the current study are available from the corresponding author on reasonable request.

## Abbreviations

KPro: Boston type I keratoprosthesis
KPro + PPV + SOI: Boston type 1 keratoprosthesis combined with pars plana vitrectomy and silicone oil insertion
BCVA: Best-corrected visual acuity
RPM: Retroprosthetic membrane
PPV: Pars plana vitrectomy
SOI: Silicone oil insertion
SLUEI: SAINT Louis University Eye Institute
GDI: Glaucoma drainage implant.
